# Male–male social bonding, coalitionary support and reproductive success in wild Guinea baboons

**DOI:** 10.1098/rspb.2022.0347

**Published:** 2022-05-25

**Authors:** Federica Dal Pesco, Franziska Trede, Dietmar Zinner, Julia Fischer

**Affiliations:** ^1^ Cognitive Ethology Laboratory, German Primate Center, Göttingen, Germany; ^2^ Leibniz ScienceCampus Primate Cognition, Göttingen, Germany; ^3^ Primate Genetics Laboratory, German Primate Center, Leibniz Institute for Primate Research, Göttingen, Germany; ^4^ Department for Primate Cognition, Georg-August-University Göttingen, Göttingen, Germany

**Keywords:** male–male relationships, social bonds, coalitionary support, reproductive success, *Papio papio*

## Abstract

Male–male bonds may confer substantial fitness benefits. The adaptive value of these relationships is often attributed to coalitionary support, which aids in rank ascension and female defence, ultimately resulting in greater reproductive success. We investigated the link between male–male sociality and both coalitionary support and reproductive success in wild Guinea baboons. This species lives in a tolerant multi-level society with reproductive units comprising a male and 1–6 females at the core. Males are philopatric, form differentiated, stable and equitable affiliative relationships (strong bonds) with other males, and lack a clear rank hierarchy. Here, we analysed behavioural and paternity data for 30 males and 50 infants collected over 4 years in the Niokolo-Koba National Park, Senegal. Strongly bonded males supported each other more frequently during conflicts, but strong bonds did not promote reproductive success. Instead, males that spent less time socializing with other males were associated with a higher number of females and sired more offspring. Notably, reproductively active males still maintained bonds with other males, but adjusted their social investment in relation to life-history stage. Long-term data will be needed to test if the adaptive value of male bonding lies in longer male tenure and/or in promoting group cohesion.

## 1. Introduction

According to sexual selection theory [1,2], males with higher quality should have greater reproductive success. In numerous species, males with the best fighting ability, i.e. the greatest strength or the best weapons, have advantages in male–male competition, gain higher dominance ranks and better access to fertile females, and sire the highest number of offspring [3]. A classic case are Northern elephant seals (*Mirounga angustirostris*), where the heaviest males reap the vast majority of matings [4]. Such intrasexual competition is typically more distinct in males, whereas mate choice is more prevalent in females [5,6]. Females may prefer males that have more exaggerated ornaments [1,7] or that spend more time and energy in elaborate courtship displays [8]. In group living animals, male reproductive success may not only depend on strength or ‘beauty’, but also on ‘social capital’, that is, the ability to cooperate and forge bonds with other males.

As observed in a wide range of taxa, including non-human primates, lions, horses, dolphins and some species of bird and fish, cooperation between males can aid in female defence resulting in longer tenure and/or increased number of females and offspring [9–15]. A prime example are male lions (*Panthera leo*) where larger coalitions are more successful in taking over female prides resulting in longer tenure and greater number of surviving offspring [9]. Similar mechanisms occur in some multi-level primate societies, where ‘leader males’ with associated ‘follower males’ have longer tenure, higher numbers of females and more offspring [12,13].

Enhanced reproductive success has also been linked to ‘strong bonds’ between males, defined as affiliative relationships that are differentiated, equitable and stable over time [16]. A number of studies have shown that investments in strong bonds are linked to increased coalitionary support, which in turn results in rank ascension and, ultimately, enhanced reproductive success [17–19]. In chimpanzees (*Pan troglodytes*), male siring success is also associated with the establishment of a large network of strong ties with other males [18]. In addition, male–male bonds may also affect female choice, as male coalitions may reduce harassment from other males and decrease infanticide risk [10,12,20] or offer better protection against predators [21].

We investigated the reproductive benefits of strong bonds between males in wild Guinea baboons (*Papio papio*). Guinea baboons live in a nested multi-level society, with ‘units’ composed of a ‘primary’ male, one to six associated females, and immatures at the core of the society [22]. Several units, together with ‘bachelor’ males, make up a ‘party’ and two to three parties regularly aggregate into ‘gangs’ with overlapping home ranges [23]. Most primary males (76.5%) have one or more associated bachelors and bachelor males are often (66.7%) associated with multiple units [24]. Primary males maintain largely exclusive affiliative and sexual relationships with the females in their unit, while bachelors exchange a smaller proportion of social interactions with females and are usually not reproductively active [25]. ‘Solitary’ males, as observed in hamadryas baboons [26], occur only rarely [24]. Males are predominately philopatric, display a high degree of spatial tolerance, form strong bonds and support each other in coalitions [23,24]. Strongly bonded males are on average more closely related than less strongly bonded males indicating that kin selection plays a role in male–male bonding [24]. Nevertheless, relatedness does not seem to fully explain male–male relationship patterns in our study population [22]. Compared to other baboon species, males show low rates of aggression and no clear dominance hierarchy [24], while females have high levels of spatial freedom and play an active role in the formation and maintenance of inter-sexual relationships [25].

We predicted that strong bonds between males—enhanced by coalitionary support—would result in higher male reproductive success via the attraction of more females, resulting in a higher number of offspring. To test this prediction, we determined bond strength following Dal Pesco *et al*. [24] and assessed the link between bond strength and coalitionary support. We predicted that dyads with stronger bonds would be more likely to cooperate during conflicts. Our core analysis examined whether male bond strength and the number of strong bonds a male has were linked to enhanced reproductive success in the form of increased numbers of associated females and sired offspring.

## 2. Material and methods

### (a) Field site, study subjects and data collection

Throughout the course of 45 months (April 2014–December 2017), we collected data on wild Guinea baboons—one of six baboon species [27]—at the Centre de Recherche de Primatologie (CRP) Simenti field station in the Niokolo-Koba National Park in Senegal (described in [22]). During the study period, the Simenti Guinea baboon community comprised approximately 400 individuals including five habituated parties in two gangs. The two parties with the highest number of males were selected as our study groups (party 9 from the Mare gang and party 6 from the Simenti gang). We used the party as our group unit and restricted all analyses to within-party interactions [24,28]. Party size and composition varied during the study period due to maturation, dispersal/migration and mortality with an average of 43 individuals in party 6 (range: 35–48, average adult sex ratio (male : female) of 1.03) and 46 individuals in party 9 (range: 38–51, average adult sex ratio (male : female) of 0.48).

We performed behavioural observations of all adult and all small and large subadult males belonging to the two study parties (*n* = 30; party 6, *n* = 16; party 9, *n* = 14). Males were included as focal subjects when they were first classified as small subadult (approx. 6 years old). At this age, they already establish close affiliations and display strong bonds and coalitionary support [24,29] with adult males. All details about male presence and age category changes, age category assessment and criteria for subject selection/exclusion can be found in the electronic supplementary material, appendix S1, tables S1 and S2, and figure S1. We conducted behavioural observations for a total of 872 observation days (1956 contact hours for party 6 and 1954 contact hours for party 9). All data were collected using customized electronic forms developed for our long-term data collection in Pendragon 7.2 software (Pendragon Software Corporation, USA) on Samsung Note 2 handhelds. We recorded census information about demographic changes (including birth, death, dispersal/migration and presence/absence), health status and female reproductive state [25] on every observation day. In all analyses, we controlled for the time a male was present in the study party, due to entering the subadult age category or death/disappearance.

We conducted 20 min focal follows [30] balanced between subjects and time of day, for an average of five monthly protocols per individual and a total focal time of 1547 h (total number of focal protocols = 4787). Protocols included recordings of continuous focal animal activity (i.e. moving, feeding, resting and socializing) and all occurrences of social behaviours such as approach within 1 m, retreat, grooming, contact-sit and greeting. All grooming and contact-sit durations were recorded to the nearest second. Instances of aggression, coalitionary support, copulation and grooming were additionally recorded ad libitum. Coalitionary support was scored every time two or more individuals simultaneously directed aggression toward a common target that could be a single male or another male–male coalition. Only coalitions involving two male allies against a common male target were included in our analysis. Due to the very low rate of aggression, all occurrences of coalitionary support between males, including both focal and ad libitum events, were included in our analysis.

### (b) Male–male social bonds and unit composition

We used the dyadic composite sociality index (hereafter DSI [31]) to quantify dyadic affiliative relationship strength. This index ranges from 0 to infinity with a mean of 1 and measures the deviation of affiliative behaviour of a given dyad compared to all other dyads in the same group. The DSI is calculated using the following formula:

$${\mathrm{DSI}}_{xy}=\frac{\sum_{i=1}^{d}(f_{ixy}/{\bar{\;f}}_{i})}{d},$$

where *f_ixy_* is the behavioural rate for dyad *xy* and behaviour *i*, ${\bar{\;f}}_{i}$ is the average behavioural rate for behaviour *i* calculated across all dyads in the party, and *d* is the number of behaviours included in the index calculation [31]. We computed yearly DSI values (January to December) for each male–male dyad within the party using the following positively correlated affiliative behaviours: grooming frequency and duration, contact-sit frequency and duration, and frequency of within 1 m approaches [24]. To avoid redundancies with other behaviours, only approaches that were not followed by social behaviour (positive or negative) within 10 s were considered in the DSI calculation. Individual bond strength was calculated as the sum of a male's top three DSI values [32]. The number of strong bonds per male was based on the number of higher than average DSI values [33].

Data on female–male interactions (i.e. frequency of copulations, grooming bouts, contact-sit bouts, greetings, and aggression events and duration of grooming and contact-sit bouts), unit composition and female unit transfers were recorded on every observation day. Following established methodologies [24,28] based on previous findings showing that females exchange significantly higher rates of interactions with their primary male [25], we used female–male interaction occurrence to verify daily unit composition within each study party.

### (c) Genotyping and paternity analysis

To establish paternity, we collected faecal samples of all subadult and adult males (*n* = 30) and subadult and adult females (*n* = 33) in party 6 and party 9. Fifty infants were born during the study period in these two parties. We were able to collect faecal samples from 36 infants for paternity analysis, while the remaining 14 infants deceased before sampling could occur. To check for extra-party paternities, we additionally sampled all subadult and adult males (*n* = 17) belonging to the other three habituated parties of our study population as well as two adult males that were associated with party 6 for only 36 days (see electronic supplementary material, appendix S1).

We evaluated individual allelic variation based on 24 polymorphic autosomal microsatellite markers. This microsatellite panel [34] is an optimized version of the panel that was successfully used in several studies of Guinea baboons (e.g. [35]) and our own study population [23]. Genetic sample collection, storage, DNA-extraction and genotyping methodologies are described in detail in Dal Pesco *et al*. [34]. Detailed information about number and type of samples available per individual can be found in the electronic supplementary material, appendix S2 and S3.

Following the methodologies in Dal Pesco *et al*. [34], we calculated descriptive statistics for all 24 markers (including *F*_IS_, expected and observed heterozygosity) and tested for Hardy–Weinberg equilibrium and presence of null alleles (see electronic supplementary material, table S3). All loci were polymorphic with allele numbers averaging 4.0 (s.d. = 1.4, range = 2.0 to 7.0). As locus D1s548 showed signs of null alleles, it was excluded from the paternity analysis, which was therefore performed using a total of 23 loci.

We estimated paternity using the software Cervus (v. 3.0.7) [36] and following the methodologies explained in detail in Dal Pesco *et al*. [34]. We recorded the identity of the mother during field observations and additionally checked all mother/offspring pairs with a maternity likelihood analysis (criteria for acceptance: identification as candidates with 0 mismatches). All mothers were confirmed with 0 mismatches. We then used a trio likelihood approach where the identity of the mother was known to determine the most likely father (see electronic supplementary material, table S4). A male was considered to have sired an offspring when he was assigned as the most likely father and had a maximum of one mismatched allele, and the confidence level for the assignment was more than 95% (‘strict’ criterion).

### (d) Statistical analyses and modelling

All statistical analyses and figure preparation were performed in the R environment (v. 4.0.5) [37] using the RStudio interface (v. 1.4.1106-5) [38]. We ran generalized linear mixed models (GLMM) [39] using the R packages ‘lme4’ (v. 1.1-26) [40] for all Poisson models and ‘glmmTMB’ (v. 1.0.2.9000) [41] for the beta model used in our post hoc analysis.

To reduce type I error rates, we used the maximal random effect structure comprising all theoretically identifiable random slope components [42] excluding the correlations between random intercepts and slopes when ‘unidentifiable’ (i.e. absolute correlation parameter approximately 1) [43]. To ease model conversion and estimate comparison, all covariates were z-transformed to a mean of zero and a standard deviation of one prior to fitting each model [44]. Detailed information about sample size, model complexity, checks for the need of zero inflation, random slopes, data standardization/transformation (including means and standard deviations of original values), model stability and the use of non-default optimizers can be found in the electronic supplementary material, appendix S4 and the tables S5–S10.

Before inference, all models were validated using diagnostic checks. We assessed the absence of collinearity among predictors calculating the variance inflation factors (VIF) [45] using the ‘vif’ function of the package car (v. 3.0-10) [46] on reduced general linear models with all random effect structures and optimizers excluded. With an overall maximum VIF value of 1.95, we ruled out collinearity for all our models. We evaluated the assumption of normality for each random effect component by visually inspecting histograms of each random intercept and slope. No obvious deviation from these assumptions was recorded. For all models (see details in each sub-section), we calculated the dispersion parameter to check for potential type I errors due to overdispersion.

In models with multiple predictors of interest, we first determined the significance of the full model (also including all predictors of interest) against a null model comprising only the control predictors and the random effect structure using a likelihood ratio test [47]. This allowed us to test the overall effect of our predictors of interest avoiding ‘cryptic multiple testing’ [48]. *P*-values for individual predictors were obtained using the likelihood ratio test of the ‘drop1’ R function with argument ‘test’ set to ‘chisq’ [42]. The function ‘bootMer’ of the package ‘lme4’ was used to perform a parametric bootstrap (1000 bootstraps) and obtain 95% model estimate confidence intervals. Effect sizes were calculated using the ‘r.squaredGLMM’ function of the ‘MuMIn’ R package (method Trigamma; v. 1.43.17) [49] and the ‘r2’ function of the ‘performance’ R package (v. 0.7.1) [50] for all Poisson models and the beta model used in our post hoc analysis, respectively.

### (e) Male–male sociality and coalitionary support

To investigate whether males with stronger bonds were more likely to support each other in coalitions we ran a GLMM [39] with Poisson error structure and log link function [51] where dyadic coalitionary support frequency per year (including both focal and ad libitum events) was the count response and yearly DSI (log- and then *z*-transformed) was the predictor of interest. To control for observation effort, we included the log-transformed contact time in hours as an offset term [51]. Note that contact time was calculated using the total time spent working with each study party during each daily working session and taking into account demographic changes for each male–male dyad. We included year and party membership as fixed control factors, and male identities (subject identity and coalition partner identity) and dyad identity (composed by subject identity followed by coalition partner identity) as random intercepts. The following random slope components were also included: year (manually dummy coded and then centred) and DSI (*z*-transformed) within both male identities. The model was not overdispersed (dispersion parameter = 0.289).

### (f) Male–male sociality and reproductive success

To examine if greater levels of male–male sociality were associated with enhanced male reproductive success, we analysed two different measures of male reproductive success: number of associated females and number of sired offspring. To account for unit size variation due to female transfers and demographic changes, we used daily unit size data to calculate the number of associated females within each year as a yearly mode per male (i.e. the most frequent unit size value). The number of sired offspring was calculated as the sum of sired offspring per male within each year (*n* = 49; one offspring was fathered by a male of another party; see electronic supplementary material, table S4). As within each unit paternity probability for the primary male is very high (for this dataset 91.7% of offspring were sired by the mother's primary male at time of conception), for the 14 offspring for whom we had no genetic data, we selected the mother's primary male at the time of conception as the most likely father. Our measures of male–male sociality were male bond strength, calculated as the yearly sum of a male's top three DSI values, and number of strong bonds, calculated as the yearly number of higher-than-average DSI values per male.

We ran two GLMMs [39] with Poisson error structure and log link function [51], where the yearly mode of number of associated females and yearly number of sired offspring were the count responses and male bond strength and number of strong bonds were the predictors of interest. In both models, we included year and party membership as fixed control factors and male identity as a random intercept. The following random slope component was also included in both models: male bond strength (*z*-transformed) within male identity. Both models were not overdispersed (dispersion parameters = 0.542 and 1.159).

### (g) *Post hoc* analysis: time males spent affiliating with other males by number of associated females

In light of the results of our analysis, we performed a *post hoc* investigation to look at the effect of the number of associated females on the proportion of time males spent affiliating (i.e. grooming and contact-sit) with other males. This allowed us to specifically look at male time budgets and to analyse interaction occurrence, which can in some cases represent social relationships more accurately and precisely compared to composite sociality indices [52].

We ran a GLMM [39] with a beta error structure and logit link function [51,53] with the proportion of time males spent affiliating with other males as the response and number of associated females as the predictor of interest. To avoid response values being exactly zero or one, we transformed the response prior to fitting the model using the following formula *x*^'^ = (*x* × (length(*x*) − 1) + 0.5)/length(*x*) [54]. We included year and party membership as fixed control factors, and male identity as random intercept. The model presented signs of moderate overdispersion (dispersion parameters = 1.283), which could not be resolved by specifically modelling dispersion with the argument ‘dispformula’. To adjust for overdispersion and the increase type I error rate, we corrected the estimate standard errors by the overdispersion level according to Gelman and Hill (SEadjusted = s.e. × √dispersion parameter) [55]. Furthermore, *z*- and *p*-values were determined again based on the adjusted standard error with *z* = estimate/SEadjusted and *p* = 2 × pnorm(*q* = −abs(*z*)).

## 3. Results

### (a) Male–male sociality and coalitionary support

Males maintained differentiated male–male relationships, with DSI values ranging from 0.00 to 21.03 (s.d. = 2.29; median = 0.06). About a fifth (20.7%) of the dyads had a DSI above the party average. The average bond strength per male was 9.35 (s.d. = 6.51; range = 0.27 to 33.95) and the average number of strong bonds per male was 2.18 (s.d. = 1.52; range = 0 to 6). The average DSI across all strongly bonded male dyads was 4.37, indicating that these dyads affiliated four times as often/long as compared to the average of the party.

**Figure 1.**
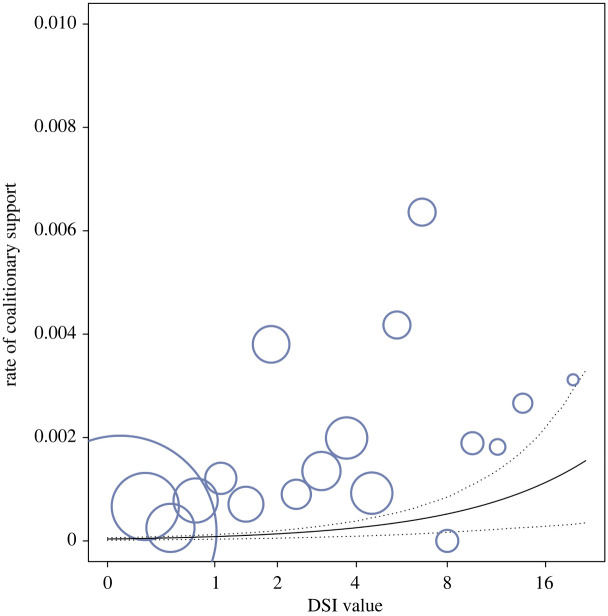
Relationship between male–male dyadic bond strength (DSI value) and dyadic rate of coalitionary support. Dyads with stronger bonds were more likely to support each other in coalitions (GLMM: *n* = 958, *p* < 0.001). DSI values are represented in log-scale and binned in 19 bins. The area of the circles depicts the frequency with which a given number of coalitions per contact hour occurred in a given bin (mean = 3.29, range = 1 to 232). The solid line depicts the fitted model and the dashed lines depict the bootstrapped 95% confidence intervals with all other predictors being at their average (party and year manually dummy coded and centred). (Online version in colour.)

A total of 290 two-against-one coalitions were recorded between males during the study duration (both from focal and ad libitum data) with 26.9% of dyads (*n* = 53 of 197) engaging in at least one coalition. Overall, dyads supported each other on average 1.47 times (s.d. = 4.78; range = 0 to 36) across the study period with an average rate per hour of 0.001 (s.d. = 0.003; range = 0.000 to 0.021) coalitions. Dyads with higher DSI values were more likely to support each other in coalitions (estimate ± s.e. = 0.781 ± 0.108, CI[0.500,0.994], *p* < 0.001, figure 1, also see electronic supplementary material, table S5).

### (b) Male–male sociality and reproductive success

**Figure 2.**
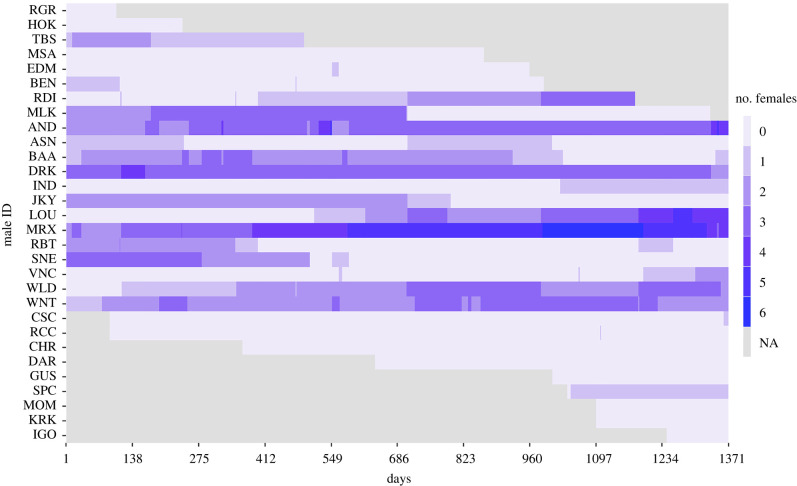
Visualization of the variation in male status and unit size (i.e. number of associated females) over the course of the study period (April 2014–December 2017) for the 30 study subjects. NA (not assessed—in grey) indicates days when males were not present due to demographical changes (i.e. not associated with the study parties, not in the selected age category, or deceased, also see electronic supplementary material, appendix S1 and figure S1). (Online version in colour.)

Twenty-one of the 30 study males had at least one associated female during part or the entire study period, while the remaining males were not associated with a female during the study period (figure 2). Of the nine males that never had primary status, seven were subadult males and two were old adult males for most of the study time during which they were present in the study party, corroborating the observation that bachelor males are often subadult or late-prime/old males [24]. Of the 21 males that had primary status at least once, 12 were adult males for their entire presence time, eight transitioned from subadult to adult during the study period, and one was a large subadult male during his presence time (see electronic supplementary material, figure S1 for male age category changes).

**Figure 3.**
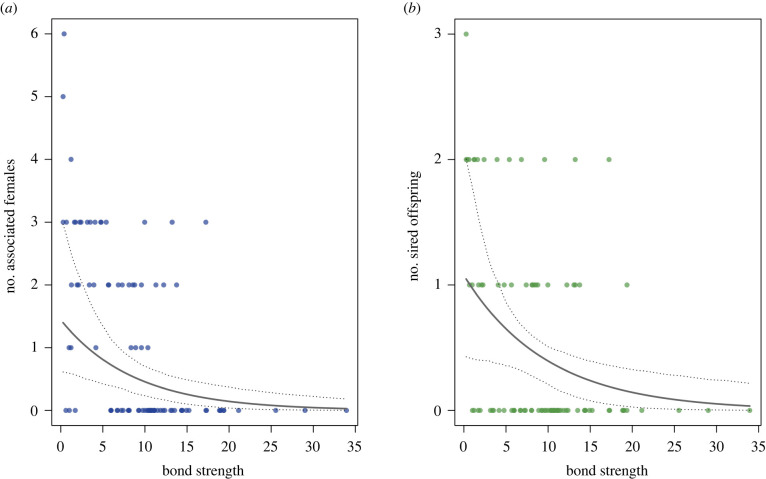
Relationship between male bond strength (calculated as the sum of a male's top three DSI values) and (*a*) number of associated females (mode per male per year) and (*b*) number of sired offspring (count per male per year). Males with stronger bonds were found to have fewer associated females (GLMM: *n* = 91, *p* = 0.003) and to sire fewer offspring (GLMM: *n* = 91, *p* = 0.017). Points represent each subject in a given year (2014–2017). The solid line depicts the fitted model and the dashed lines the bootstrapped 95% confidence intervals with all other predictors being at their average (party and year manually dummy coded and centred and number of strong bonds *z*-transformed to a mean of 0 and standard deviation of 1). (Online version in colour.)

**Figure 4.**
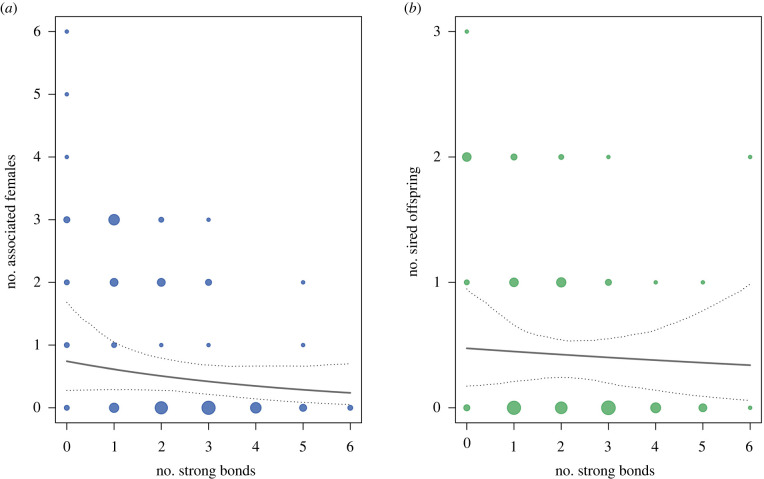
Relationship between number of strong bonds (calculated as the number of higher-than-average DSI values per male) and (*a*) number of associated females (mode per male per year) and (*b*) number of sired offspring (count per male per year). There was no evidence for a relationship between number of strong bonds and number of associated females (GLMM: *n* = 91, *p* = 0.181) or number of sired offspring (GLMM: *n* = 91, *p* = 0.727). Points represent each subject in a given year (2014–2017). The solid line depicts the fitted model and the dashed lines the bootstrapped 95% confidence intervals with all other predictors being at their average (party and year manually dummy coded and centred and number of strong bonds *z*-transformed to a mean of 0 and standard deviation of 1). (Online version in colour.)

The average mode of the number of associated females per male per year was 1.09 (s.d. = 1.40; range = 0 to 6). This average was 1.29 (s.d. = 1.43; range = 0 to 6) if we excluded males that never had primary status throughout the study period. The full model including the two predictors of interest (male bond strength and number of strong bonds) accounted for significantly more variance compared to the null model (full-null model comparison: *χ*^2^ = 22.237, *p* < 0.001). While there was no obvious evidence that the number of strong bonds had an effect on the number of associated females (estimate ± s.e. = −0.288 ± 0.220, CI [−0.779, 0.176], *p* = 0.181), we found strong evidence that males with higher bond strength were associated with fewer females (estimate ± s.e. = −0.749 ± 0.266, CI [−1.381, −0.254], *p* = 0.003) (see figures 3*a* and 4*a*, also see electronic supplementary material, table S6). The negative effect of male bond strength held true when we analysed a subset of data only including adult and large subadult males within each year (estimate ± s.e. = −0.739 ± 0.259, CI [−1.368, −0.281], *p* = 0.002; see electronic supplementary material, table S9).

Overall males in the study parties sired 49 offspring (one offspring was fathered by a male of another party) with an average number of 1.63 (s.d. = 2.13; range = 0 to 8) offspring sired per male across the study period. The average number of offspring sired across the study period was 2.33 (s.d. = 2.20; range = 0 to 8) if we only considered males that had primary status at some point during the study period. The average number of offspring sired per male per year was 0.54 (s.d. = 0.78; range = 0 to 3). This average was 0.64 (s.d. = 0.81; range = 0 to 3) if we excluded males that never had primary status throughout the study period. The full model with the two predictors of interest (male bond strength and number of strong bonds) accounted for significantly more variance compared to the null model (full-null model comparison:$\;\chi^{2}$ = 11.260, *p* = 0.004). While there was no obvious evidence that number of strong bonds had an effect on the number of sired offspring (estimate ± s.e. = −0.085 ± 0.245, CI [−0.629, 0.367], *p* = 0.727), we found moderate evidence that males with higher bond strength sired fewer offspring (estimate ± s.e. = −0.655 ± 0.288, CI [−1.358, −0.144], *p* = 0.017) (see figures 3*b* and 4*b*; also see electronic supplementary material, table S7). The negative effect of male bond strength held true when we analysed a subset of data only including adult and large subadult males within each year (estimate ± s.e. = −0.664 ± 0.289, CI [−1.372, −0.151], *p* = 0.013; see electronic supplementary material, table S10).

### (c) *Post hoc* analysis: effect of number of associated females on time spent affiliating with other males

**Figure 5.**
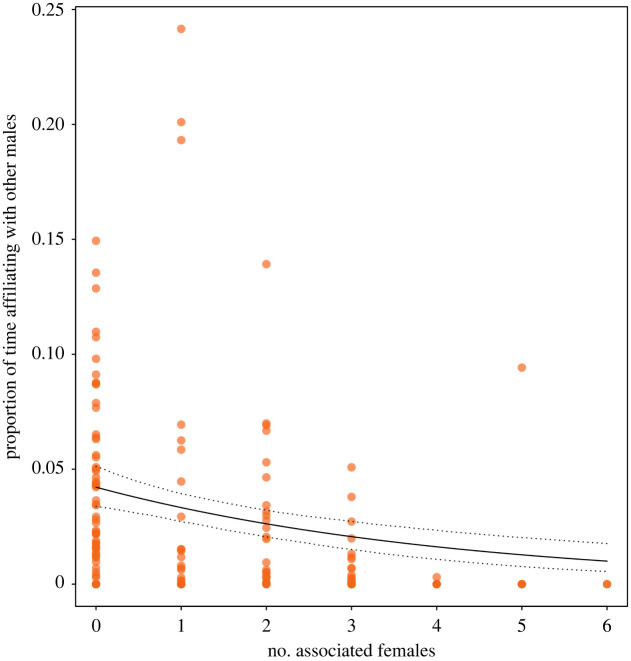
Effect of the number of associated females per male on the proportion of time males spent affiliating with other males. Males with higher numbers of associated females spent lower proportions of time affiliating with other males (GLMM: *n* = 147, *p* < 0.001). Points represent each dyad in a given year (2014–2017). The solid line depicts the fitted model and the dashed lines depict the bootstrapped 95% confidence intervals with all other predictors being at their average (party and year manually dummy coded and centred). (Online version in colour.)

Contrary to our predictions, male bond strength was linked to lower numbers of associated females. We therefore performed a *post hoc* analysis focused on male time budget to explore the relationship between time spent affiliating with other males and the number of associated females. We found strong evidence that males with higher numbers of associated females spent a lower proportion of time affiliating with other males (estimate ± s.e. = −0.371 ± 0.107, CI [−0.550, −0.209], *p* < 0.001; figure 5; see electronic supplementary material, table S8).

## 4. Discussion

Contrary to our predictions, we found no evidence that male–male sociality was linked to higher reproductive success. Instead, we observed a strong negative relationship between bond strength and male reproductive success (i.e. number of associated females and paternities). Guinea baboon males that were associated with a higher number of females spent less time affiliating with other males. While a number of now classic studies reported a positive relationship between sociality and reproductive success in both males and females across several mammalian [17,18,32,56] and bird species [14], our results indicate that male–male sociality need not directly translate into increased short-term reproductive success. Instead, males that invest time and energy in relationships with females, at the expense of relationships with males, have the highest reproductive success.

Interestingly, reproductive success was not obviously negatively related to the number of strong bonds a male had, indicating that males do maintain differentiated relationships with other males, but mainly adjust their time budgets in relation to the number of females they are able to attract. As inter-sexual bonding patterns in this species are largely driven by female choice [25] and in light of the high paternity certainty within units (91.7% in this study), stable bonds with females confer direct fitness benefits. It therefore pays for males to invest in bonds with females, irrespective of their reproductive state [25]. Similar patterns were observed in horses (*Equus caballus*), where less successful stallions maintained stable alliances with others, while successful ones exclusively focused on their mares [10].

Guinea baboon males appear to face a trade-off between investments in same-sex and opposite-sex bonds, and the investment in different types of bonds varies with life-history stage: young and old bachelor males invest more in same-sex relationships, but turn their attention to females once they have become primary males—at the expense of time available for their male ‘friends’. Similar effects are seen in male Barbary macaques (*Macaca sylvanus*) and snub-nosed monkeys (*Rhinopithecus bieti*) across seasons, where investment in male—male affiliative relationships drops during the mating season [57,58].

Long-term data will be needed to assess whether male–male bonds are related to an earlier/later acquisition of females, thus increasing tenure length and in this way reproductive success. Additionally, bonds may constitute a ‘fall-back’ option for males once they lose their status as a primary male by providing support and tolerance in old age, and indirectly promoting group cohesion. Indeed, Barbary macaque males rely more heavily on cooperative strategies during their post-prime phase [59], while older chimpanzees show greater levels of positive behaviours as well as higher numbers of mutual male–male relationships [60]. For now, we are confident that male–male sociality is negatively linked to reproductive success over the short term, but cannot exclude the possibility that bonds increase lifetime reproductive success via earlier or longer male tenure.

How do males manage their relations with other males, when most of their social investments go to females? Under time budget constraints, Guinea baboon males may use male–male ritualized greeting behaviour, characterized by quick, stylized and costly exchanges [28], to assess and maintain their relationships. We propose that the most intense and potentially costly forms of greetings, which occur more often between strongly bonded males [28], can play a central role in male–male bond maintenance once males acquire primary status and invest less time in affiliation. Similarly, in macaques, ritualized interactions between males have been proposed as efficient means in maintaining bonds when their social time with other males is limited [61].

Regarding coalition formation, Guinea baboon males with stronger bonds supported each other more often during agonistic events, corroborating previous analyses in the same [23] and several other species [17,18,57,62]. Compared to macaques, however, rates of coalitionary support in Guinea baboons are low (0.001 h^–1^; Assamese macaques, *Macaca assamensis*: 0.11 h^–1^ [17]; Barbary macaques: 0.01–0.21 h^–1^ [57]), mirroring the low rate of aggression [24]. Given the lack of a clear dominance hierarchy between males [24] and the presence of frequent instances of coalitions targeting other coalitions [29], it is unlikely that coalitions serve in rank ascension. Why Guinea baboon males engage in possibly risky coalitions and what benefits strong bonds and cooperation may confer requires further investigation.

Ultimately, Guinea baboon females may not gain much from preferring males with strong bonds, as males rarely attempt to takeover females from other males, and infanticide has not been observed in this population [22,25]. Moreover, females do not appear to choose males with strong bonds as a means of protection from predators [21]. Instead, females may simply prefer males in good condition. Indeed, mane coloration and length, as well as hind-quarter coloration, have been proposed as honest signals of male quality in hamadryas and Guinea baboons [63,64], a hypothesis that remains to be tested. In male geladas, redder chest patches are associated with higher status and larger units [65]. Our current working hypothesis is that male condition and attention to the female are the key determinants of female choice. Although females may have preferences for specific males, female benefits may decrease in larger units due to higher levels of female–female competition over social support and mating opportunities [66]. Female choices are therefore likely also affected by the size and composition of the unit. Considering that males sometimes show parental care (pers. observation) and are generally tolerant toward females, it is also possible that females take into account a male's willingness to provide care for offspring, as reported in mountain gorillas [67], or a male's disposition to accept females' spatial freedom [68]. Long-term data will be needed to test these ideas.

Taken together, we suggest that female choice explains male–female associations, while female–female competition may result in an upper limit on unit size. Consequently, almost all males in their prime achieve some reproductive success and there is little to fight over. Variation in male–male sociality may thus be conceived as an outcome of males adjusting their affiliation patterns according to females’ choices. Our study reinforces the view that male strategies may vary considerably in relation to female leverage in mate choice, and that even among closely related species, such as in the genus *Papio*, entirely different strategies may evolve. Our findings add a piece to the puzzle of understanding the co-evolutionary dynamics of male and female strategies.

## Data Availability

The data that support the findings of this study are openly available on OSF as 'Data and scripts for male–male social bonding, coalitionary support and reproductive success in wild Guinea baboons' via this link: https://osf.io/7v8r5/?view_only=fbfba4deb6284522b67d955b91d902df. Electronic supplementary material is available online [69].
